# Supplementary material for: Copy number variations and their effect on the plasma proteome
Source: Genetics. 2023 Oct 4;225(4):iyad179. doi: 10.1093/genetics/iyad179 (PMC10697815; doi:10.1093/genetics/iyad179)
Supplement: iyad179_Supplementary_Data [file iyad179_supplementary_data.zip › Data access policy.docx]

# Procedure for requesting access

Access to pseudonymized individual level data for NSPHS for research purposes must be requested in writing from corresponding author Åsa Johansson (asa.johansson@igp.uu.se).

Data access requires that an application has been approved by the Swedish Ethical Review Authority (EPM), for which comprehensive information and a digital application portal are available at (<https://etikprovningsmyndigheten.se/>).

The application must have a clear scientific purpose and clearly describe the objectives, methods, timetable, data management, and ethical considerations, as well as details about the principal investigator and all collaborators that needs to have access to the data, together with information about the entity responsible for the principal investigator.

# Parties responsible for evaluating requests

Åsa Johansson, EPM

# Criteria and procedure used to evaluate requests

The research project must be approved by EPM who will also evaluate whether data access shall be granted. Upon reception of a data access request with an application approved by EPM and expressly allowing access to NSPHS individual-level data, the corresponding author will grant data access.

# Conditions for data access

1. Data delivery is subject a material transfer agreement (MTA) between Uppsala University and the receiving entity.
2. The requesting party must guarantee confidentiality of the data and adhere to the General Data Protection Regulation (GDPR).
3. Results from the research project must not be used nor licensed for commercial purposes.
4. Results from the proposed research project must be shared with the granting parties prior to publication. The requesting party must accommodate all reasonable changes proposed by the granting parties.
5. The requesting party may not enter an agreement regarding sharing of the data without written consent from the granting parties.

# Expected response time to requests for data access

Any questions regarding data access can expect a response from the corresponding author within a week.

# Usage or types of requests that would lead to denial of access

1. The requesting party cannot guarantee confidentiality of the data.
2. Results from the research project will be patented or used/licensed for commercial purposes.
3. The proposed research project was rejected by the Swedish Ethical Review Authority
