# Supplementary material for: Copy number variations and their effect on the plasma proteome
Source: Genetics. 2023 Oct 4;225(4):iyad179. doi: 10.1093/genetics/iyad179 (PMC10697815; doi:10.1093/genetics/iyad179)
Supplement: iyad179_Supplementary_Data [file iyad179_supplementary_data.zip › Supplemental_Figures_GENETICS-2023-306425.docx]

Supplementary Figures


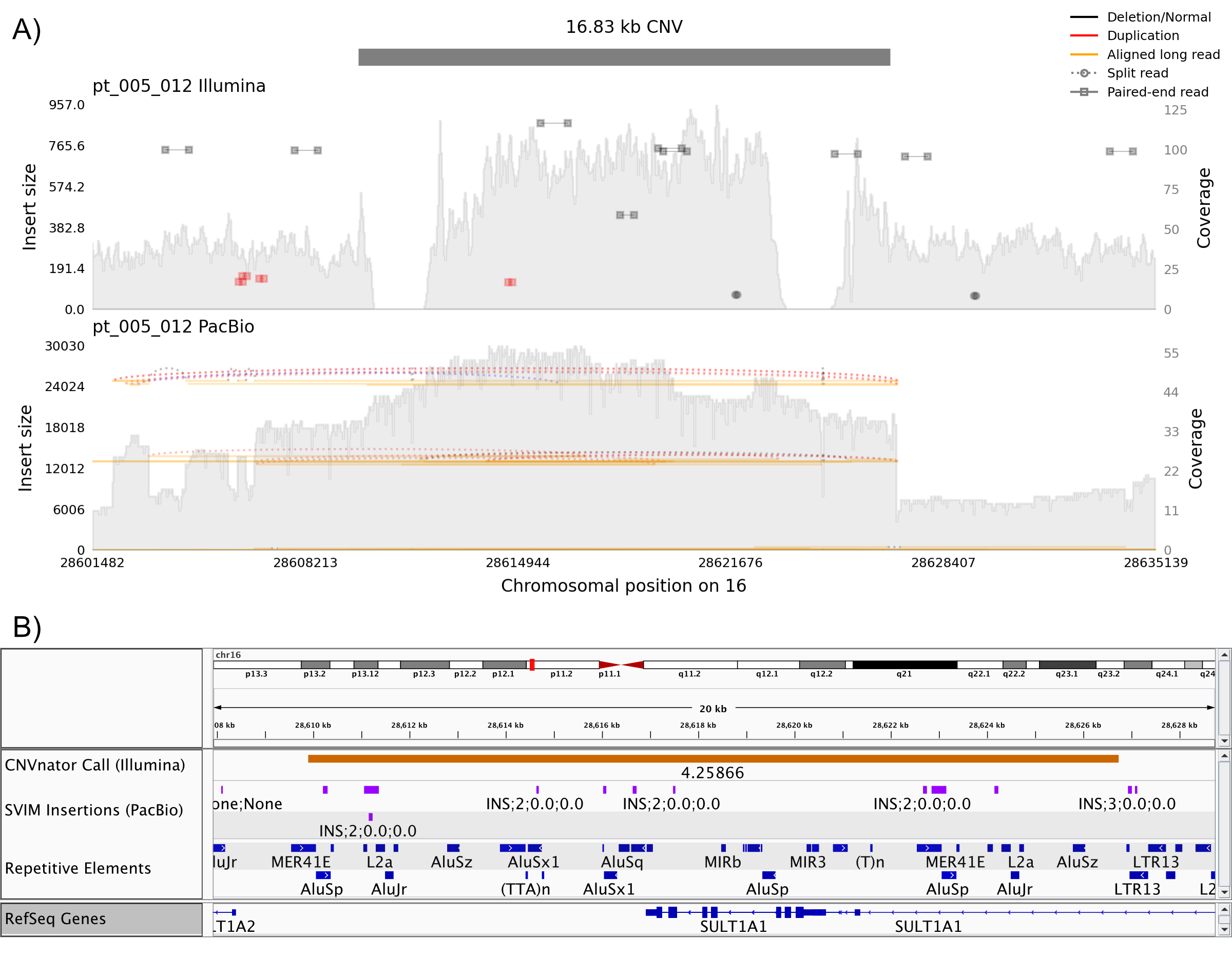


Supplementary Figure S1: **A)** Coverage plots of CNVR 12 on chromosome 16 in one individual. In this individual, this CNV was called as a duplication by CNVnator, which is illustrated by the higher coverage. There was evidence for multiple smaller duplications in this region but none mapped well to CNVnator’s call. **B)** Repetitive elements and insertions called by SVIM in the same individual. The top shows the length of the current region and its coordinates. The track CNVnator Call (Illumina) (orange) shows the CNV as called by CNVnator in that individual along with the called CN. Below, the track SVIM insertions (PacBio) (purple) shows the insertions called by SVIM from the SMRT data. The track Repetitive Elements (blue) lists annotations from RepeatMasker in that area. The bottom track shows RefSeq Genes. The insertions called by SVIM in the long reads match well with known RepeatMasker annotations. This suggests that CNVnator picked up these small insertions and merged them into one CNV call.


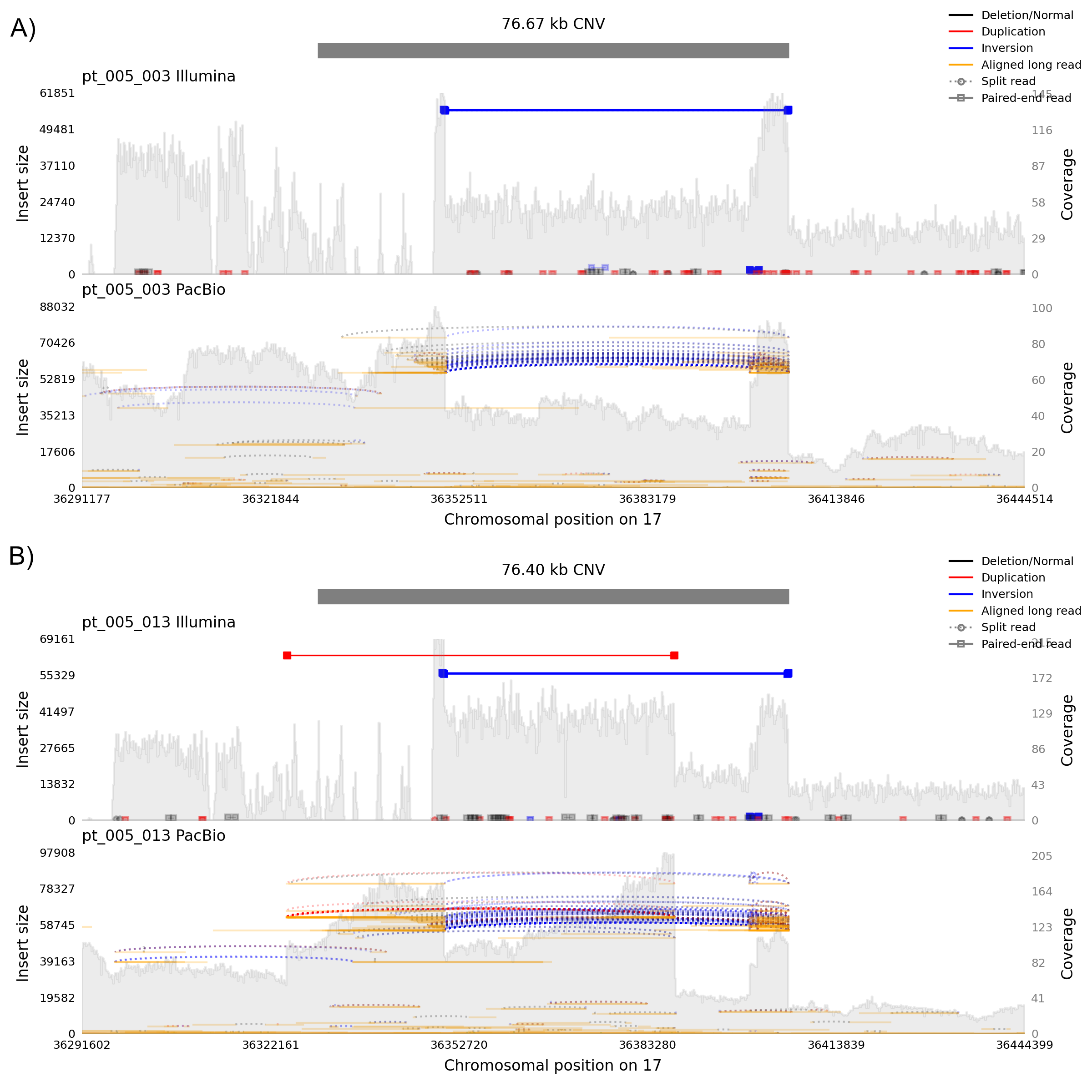


Supplementary Figure S2: Coverage plots of CNVR 13 in one individual. The top panel shows Illumina, the bottom SMRT data. The gray area represents the per-base coverage in the area. Lines in the plot show read-level evidence for SV. **A)** CNVnator called a large duplication in this region. However, the SMRT data, and to some extent the Illumina data, show evidence for a large inversion. B**)** The common inversion overlaps with a duplication, showing a characteristic pattern.
